# Leader Humor and Followers’ Change-Oriented Organizational Citizenship Behavior: The Role of Leader Machiavellianism

**DOI:** 10.3390/bs12020022

**Published:** 2022-01-25

**Authors:** Yongjun Choi, Sun-Bok Ha, Dongwon Choi

**Affiliations:** 1 College of Business Administration, Hongik University, Seoul 04066, Korea; yongjun.choi@hongik.ac.kr; 2 College of Law and Politics, Kyungnam University, Changwon 51767, Korea; 3 Ewha School of Business, Ewha Womans Univeristy, Seoul 03760, Korea; dwchoi@ewha.ac.kr

**Keywords:** humor, leader humor, team commitment, change-oriented organizational citizenship behavior, Machiavellianism

## Abstract

The purpose of this study is to explore the mechanisms by which leader humor affects followers’ change-oriented organizational citizenship behavior. Specifically, we examine the mediation effect of team commitment in the leader humor–change-oriented organizational citizenship behavior link and whether it varied by leader Machiavellianism. Using multi-sourced data from the four battalions of the Republic of Korean Army, our findings show that team commitment mediated the positive relationship between leaders’ affiliative humor and followers’ change-oriented organizational citizenship behavior. Furthermore, the mediated relationship was stronger when leader Machiavellianism was lower. On the other hand, we found no support for the negative relationship between leaders’ aggressive humor and followers’ change-oriented organizational citizenship behavior. Theoretical and practical implications are discussed.

## 1. Introduction

Employees’ proactive behaviors at work are vital for innovation in organizations [1]. Previous studies have shown that leadership plays an important role in facilitating employees’ proactive behaviors [2,3,4]. More recently, a growing body of research has emphasized the role of leaders’ humor at work as an important way of cultivating followers’ innovative and creative thinking [5,6]. Among many proactive behaviors critical for workplace innovation, change-oriented organizational citizenship behavior has emerged as an important dimension of performance, expanding the scope of organizational citizenship behavior to better account for the growing competition, uncertainty, and turbulent changes in the dynamic business environment [7]. In addition, because it captures not only one’s attitudes toward innovation and organizational changes [8], but also one’s initiative toward solving work-related problems proactively and progressively, the voice that calls for change and makes a constructive effort to improve the organization’s functionality [7] can energize innovation at work. Therefore, exploring the mechanisms by which leaders’ humor affects followers’ change-oriented organizational citizenship behavior is a valuable avenue of inquiry.

The literature on humor suggests there are several humor styles; most studies characterize humor as either positive or negative in nature [9,10]. Past studies have generally demonstrated the beneficial effects of a leader’s positive humor at work. For example, a meta-analysis by Mesmer-Magnus et al. [10] demonstrated that leaders’ positive humor tends to increase the followers’ job performance and satisfaction with their leaders, and tends to decrease work withdrawal. In a similar vein, a recent meta-analysis by Kong et al. [11] showed that leader humor increases the followers’ job performance, organizational citizenship, and job satisfaction. In addition, the analysis showed that the followers’ trust in their leaders and the leader–member exchange play an important role in mediating the positive relationship between leader humor and the followers’ attitudes and behaviors at work. In evaluating the relationship between leader humor and followers’ innovation-relevant behaviors at work, Csikszentmihaly [12] argued that leader humor could cultivate followers’ innovative thinking at work. More recently, studies have begun exploring the mechanism by which leader humor affects the followers’ innovation-relevant behaviors. Past studies demonstrated that leader humor increases the followers’ creativity via increases in relational energy [6], work engagement [13], and task resources [14]. However, our knowledge about the mechanism by which leader humor is related to followers’ change-oriented organizational citizenship behaviors is still in a nascent state.

In this study, we aim to advance our understanding of the roles of leader humor at work by elucidating the mechanism by which leader humor leads to the followers’ change-oriented organizational citizenship behaviors. First, we propose that leader humor could either increase or decrease the followers’ change-oriented organizational citizenship behaviors. Although the relationship between leader humor and organizational citizenship behaviors is relatively well established in the field [11,15], its relationship with other forms of extra-role behaviors, such as creativity [5,6,14] and job crafting [16], have just started to emerge in the humor literature. Because leader humor could provide employees with resources to engage in proactive work behaviors, including taking the initiative or making a voice for their team’s interests and success, we propose that the extent to which employees engage in change-oriented organizational citizenship behaviors could vary by the forms of leader humor (i.e., affiliative vs. aggressive humor).

Second, we propose that the followers’ team commitment, which refers to the relative strength of identification and involvement that followers perceive about their team [17,18], is a mediator that explains the relationship between leader humor and change-oriented organizational citizenship behavior. Team commitment may serve as a mediator because leader humor could encourage followers’ feelings of connectedness or disconnectedness with their team members [19], which could affect their perceptions of being identified with and involved in their teams. Extending the previous research on the process by which leader humor affects the followers’ innovation-relevant behaviors at work, an examination of the mediating role of team commitment will broaden our knowledge of the function of leader humor in facilitating innovation at work.

Third, our study incorporates leader Machiavellianism, which refers to “a duplicitous interpersonal style, characterized by a cynical disregard for morality and a focus on self-interest and personal gain” [20], into our model. Because leader Machiavellianism could affect the way that followers interpret the motives behind leaders’ humor, we propose that leader Machiavellianism moderates the mediated process. Although past studies on humor have explored the roles of personality, such as by using the big five personality model [21,22,23], relatively little attention has been paid to the dark triad personality. Exploring the moderating role of leader Machiavellianism in the leader humor–change-oriented organizational citizenship behavior realm will enrich our knowledge by answering calls for discovering a moderating influence in the literature on humor [10]. From a practical point of view, our study will help managers understand that, to energize the followers’ change-oriented organizational citizenship behavior, which is critical to achieve innovation in dynamic business environments, they are encouraged to use positive forms of humor (e.g., affiliative humor) in an appropriate manner so that their followers perceive the humor as conveying the leaders’ genuine support.

## 2. Hypotheses’ Development

In this study, first, we propose that leader humor style (i.e., affiliative vs. aggressive humor) affects a follower’s team commitment. Second, we propose that there is a positive effect of team commitment on change-oriented organizational citizenship behavior. Accordingly, we theorize that team commitment mediates the relationship between leader humor style and change-oriented organizational citizenship behavior. Finally, we propose that the mediation effect of team commitment is moderated by leader Machiavellianism.

### 2.1. Leader Humor and Team Commitment

A leader’s humor can lead to either positive or negative consequences at work. Leaders can use either positive or negative forms of humor in relating to their employees. Whereas the use of affiliative humor (a positive form of humor) enhances relationships with others, the use of aggressive humor (a negative form of humor) is detrimental to relationships [9]. Where affiliative humor is used to amuse others or facilitate the relationship quality, aggressive humor can involve sarcasm, teasing, or ridicule.

We propose that leader humor can either increase or decrease the followers’ team commitment. First, because a leader’s affiliative humor is not mandatory in a workplace, the followers may perceive their leaders’ affiliative humor as offering support and expressing friendliness toward them, and as being indicative of their willingness to build quality relationships with followers [24]. According to social exchange theory [25], followers who perceive supervisory support are more likely to reciprocate by showing high levels of commitment. Past studies also suggest that leaders’ positive humor increases the followers’ affective organizational commitment [9,15,26,27,28]. A recent meta-analysis by Kong et al. [11] also provides support for this connection by demonstrating increases in leader–member exchange (LMX), which is consistent with previous findings regarding the positive relationship between a leader’s use of affiliative humor and the followers’ perceptions of LMX [29,30,31].

Beyond affective organizational commitment, we propose that a leader’s affiliative humor increases the followers’ team commitment. Team commitment refers to “the relative strength of an individual’s identification with and involvement with the team members in their work environment” [17]. A leader’s affiliative humor is likely to allow the followers to feel connected within a team as well as increase their social membership through more frequent social interactions within those teams [19]. Furthermore, the high levels of LMX that result from a leader’s use of affiliative humor are likely to facilitate the followers’ commitment toward their teams as a way of reciprocating their leaders’ favorable treatment by pursuing goals and values that help the leaders to reach their professional goals [32,33].

Second, and in contrast to our prediction about a leader’s affiliative humor, we propose that a leader’s use of aggressive humor is likely to lessen the followers’ team commitment. A leader’s aggressive humor can signal that the leader is not willing to build and develop quality relationships with the followers [30]. Moreover, the followers may even perceive that the leader is attempting to create social distances between them [34], thereby triggering the followers’ negative emotions [35] and leading to decreased team commitment. Thus, we hypothesized the following:

**Hypothesis** **1.** *A leader’s affiliative humor is positively related to a follower’s team commitment*.

**Hypothesis** **2.** *A leader’s aggressive humor is negatively related to a follower’s team commitment*.

### 2.2. Team Commitment and Change-Oriented Organizational Citizenship Behavior

Field theory [36] suggests that the proximity and salience of environmental elements play a significant role in determining an individual’s responses to the environment [37]. Thus, it is likely that the teams—rather than the organizations—to which the followers belong more strongly affect their organizational lives [38]. Indeed, regarding levels of commitment, the followers’ team commitment is often higher than their organizational commitment [39].

Organizational commitment reflects an employee’s attachment to their organization and even their willingness to sacrifice for the organization; this commitment, in turn, is vital for prosocial behaviors [40]. Using a wide range of samples, such as university students and employees [41], major national financial institutions [42], and government organizations [43], previous research supports the positive relationship between organizational commitment and organizational citizenship behavior, which is also one form of prosocial behavior at work. For example, Kang and Lee [44] demonstrated that the more employees are committed to their organizations, the more they are likely to engage in organizational citizenship behaviors because organizational commitment helps employees to behave in accordance with organizational goals.

Followers who are committed to their teams are more likely to engage in organizational citizenship behavior because, as previously mentioned, team commitment is highly correlated with organizational commitment [17,45,46]. Past studies also suggest that employees’ team commitment has a positive effect on their organizational citizenship behavior [38,47] because, when team members are immersed in the goals and values of their teams, they become more emotionally attached to their teams and their fellow members, which, in turn, helps them to behave in a manner that will ultimately benefit their teams [48].

Expanding on previous research, in this study we expect that followers who are committed to their teams are more likely to engage in change-oriented organizational citizenship behavior. Change-oriented organizational citizenship behavior captures “[one’s] constructive efforts to bring about improvement” [49]. Because organizational commitment is motivational [50], followers who are committed to their organizations develop a sense of belonging to those organizations [51] and increase their willingness to exceed their formal responsibilities at work [52,53], which, in turn, motivates them to engage in change-oriented organizational citizenship [54,55]. In a similar vein, because followers who are more committed to their teams identify more with their teams and are more involved with them [17,18], they are more likely to take initiative in proactively improving work processes to contribute to their teams’ success. In other words, commitment to a team could help the followers to see their team’s success or failure as their own success or failure; this, in turn, leads to change-oriented organizational citizenship behaviors, including taking initiative or being a voice for their team’s interests and success. Therefore, we hypothesized the following:

**Hypothesis** **3.** *A follower’s team commitment is positively related to their change-oriented organizational citizenship behavior*.

### 2.3. Team Commitment as a Mediator

Past studies on humor have demonstrated that a leader’s humor can affect the followers’ organizational citizenship behavior. For instance, Cooper et al. [15] drew upon social exchange theory [25] in reasoning that followers whose leaders frequently use positive humor at work are more likely to engage in organizational citizenship behavior due to the increases in leader–member exchanges. Their results imply that leaders’ humor serves to reduce the social distance between leaders and their followers [24], which increases the quality of their relationship and, in turn, encourages followers to make extra efforts at work in ways that will benefit their leaders, colleagues, and organizations. In a similar vein, Goswami et al. [56] showed that a leader’s positive humor increases the followers’ organizational citizenship behavior. A recent meta-analysis by Kong et al. [11] also indicates that leader humor, especially leader humor expression, leads to increases in the followers’ organizational citizenship behavior.

Based on past findings and our current hypotheses, we expect that leader humor will affect the followers’ change-oriented organizational citizenship behavior via the followers’ team commitment. Change-oriented organizational citizenship behavior is an employee’s discretionary behavior for changes at work that could be somewhat risky from the employees’ perspectives [7,54,57]. Hence, taking such initiatives at work is largely influenced by the employees’ perceptions of the formal as well as informal work context [49]. The relational model [24] and wheel model of humor [58] suggest that leaders’ positive humor triggers the followers’ positive emotions and leads followers to judge their social environments more favorably and perceive their leaders as more supportive, resulting in positive work attitudes and behaviors [11,24]. The meta-analysis of Chiaburu et al. [59] also demonstrated that supervisor support is one of the main sources of followers’ change-oriented organizational citizenship behavior. Thus, it is likely that leaders’ affiliative humor facilitates the followers’ change-oriented organizational citizenship behavior because followers are more committed to their teams and genuinely care about their team’s success—an outcome that is a direct product of their supervisor’s support (i.e., affiliative humor). Likewise, leaders’ aggressive humor is likely to be detrimental to the followers’ change-oriented organizational citizenship behavior because it could decrease the followers’ concern for the team’s success due to their perception of a less supportive or negative supervisor. Therefore, we hypothesize the following:

**Hypothesis** **4.** *A follower’s team commitment mediates the positive relationship between a leader’s affiliative humor and the follower’s change-oriented organizational citizenship behavior*.

**Hypothesis** **5.** *A follower’s team commitment mediates the negative relationship between a leader’s aggressive humor and the follower’s change-oriented organizational citizenship behavior*.

### 2.4. Leader Machiavellianism as a Moderator

A leader’s humor at work can be perceived as either genuine or manipulative in purpose. In other words, followers might put contrasting interpretations on the underlying motives of their leader’s humor. In this study, we propose that leaders’ Machiavellianism affects the way followers view their leaders’ humor. Past studies have shown that Machiavellians are more likely to engage in impression management [60] because they are politically oriented and, thus, highly motivated to exert control over their followers for their benefit [61,62]. Machiavellian leaders are likely to engage in destructive behaviors, such as abusive supervision [63].

Followers likely form somewhat negative attitudes toward Machiavellian leaders. For example, a recent meta-analysis showed that because Machiavellian leaders are highly motivated by their own ultimate interests, they are low in empathy [64]. In a similar vein, Frazier and Jacezko [65] showed that followers who have Machiavellian leaders have low perceptions of their leaders’ ethical leadership. More generally, Jones and Paulhus [66] demonstrated that Machiavellians are perceived more negatively by others, who may regard them as unfavorable friends or business partners. Hence, even when Machiavellian leaders exhibit ostensibly affiliative humor at work, the followers are likely to interpret that humor as a deceptive behavior aimed at furthering the leader’s own interests. When Machiavellian leaders exhibit aggressive humor at work, it will likely reinforce the followers’ negative attitudes toward them. In sum, we predict that the impact of a leader’s humor style on the followers’ team commitment will vary depending on the leader’s level of Machiavellianism. Specifically, the positive impact of the leaders’ affiliative humor on the follower’s team commitment will be weakened as a result of the leaders’ high Machiavellianism; this, in turn, will decrease the followers’ change-oriented organizational citizenship behavior. In addition, when leaders’ Machiavellianism is high, the negative impact of the leaders’ aggressive humor on the followers’ team commitment will be enhanced; this, in turn, will decrease the followers’ change-oriented organizational citizenship behavior. Thus, we hypothesize the following:

**Hypothesis** **6.** *The strength of the mediated relationship between affiliative humor and change-oriented organizational citizenship behavior (via team commitment) varies based on leader Machiavellianism; the indirect effect of affiliative humor via team commitment on change-oriented organizational citizenship behavior will be weaker for those with high leader Machiavellianism than for those with low Machiavellianism*.

**Hypothesis** **7.** *The strength of the mediated relationship between aggressive humor and change-oriented organizational citizenship behavior (via team commitment) varies based on leader Machiavellianism; the indirect effect of aggressive humor via team commitment on change-oriented organizational citizenship behavior will be stronger for those with high leader Machiavellianism than for those with low Machiavellianism*.

## 3. Methods

### 3.1. Participants and Procedures

To test the hypotheses, we surveyed four battalions of the Republic of Korea Army. We surveyed squad leaders and members. Anonymity was ensured via the distribution of sealed envelopes along with the survey. The survey was delivered to a total of 393 members and their leaders. Some survey responses were not usable because of missing or incomplete answers, so the final sample included 380 members and their leaders. All subjects gave their informed consent for inclusion before they participated in the study. In addition, this study was conducted in accordance with the Declaration of Helsinki of 1975.

The followers’ average age was 22 years old, and 82% of them were college graduates. The squad leaders’ average age was 24 years old, and 89% of them were college graduates. The followers’ ranks were composed of private (9.5%), private first class (45.6%), corporal (29.15%), and sergeant (15.8%). Squad leaders included sergeant (72.8%) and staff sergeant (27.2%). The troop types included infantry (17.7%), artillery (33.5%), engineer (25.9%), and communication (22.8%).

### 3.2. Measurement

The survey items in English were translated into Korean using the conventional method of forward and back translation [67] by two bilingual (English–Korean) academics to assure equivalence. Leader’s humor style and Machiavellianism were rated by leaders (i.e., squad leaders), and the remainder of the variables, such as team commitment and change-oriented organizational citizenship behavior, were rated by followers (i.e., squad members). Because the measurement by Martin et al. [9] was intended to be self-report data, this study used multiple sources of data collection.

Study variables were assessed using multi-item scales used in previous research with acceptable internal consistency. All items were measured on a 7-point Likert-type scale ranging from 1 (strongly disagree) to 7 (strongly agree).

*Leader humor style.* To measure leader humor style, we used the Humor Styles Questionnaire (HSQ) by Martin et al. [9]. Although the HSQ consists of four dimensions of humor (affiliative, self-enhancing, aggressive, and self-defeating), given our study context focusing on the effect of humor styles on interpersonal relationships with others [9], we measured only two categories of humor style: affiliative and aggressive. Each humor style consisted of eight items. We used the 16-item scale from Martin et al. [9]. A sample item for affiliative humor is, “I enjoy making people laugh,” and a sample item for aggressive humor reads, “If someone makes a mistake, I will often tease them about it.”

*Machiavellianism.* Leaders assessed their Machiavellianism using the four-item scale from Jonason and Webster [68]. Sample items are “I tend to exploit others towards my own end” and “I tend to manipulate others to get my way.”

*Team Commitment.* Team commitment was measured using the four-item scale from Schippers et al. [69]. Sample items include “I feel proud to belong to this team” and “I am glad I belong to this team and not to another team.”

*Change-Oriented Organizational Citizenship Behavior*. We measured change-oriented organizational citizenship behavior using the four-item scale from Choi [57]. Sample items are “I frequently come up with new ideas or new work methods to perform my task” and “I often suggest work improvement ideas to others.”

*Control Variables.* To avoid influencing the results, the followers’ conflicts with their leader were controlled. Interpersonal conflicts can have a positive or negative effect on a team or team member. Although constructive conflicts may stimulate innovative thinking and the creation of new ideas [70,71], we acknowledge that conflict can also lead to tension and hostility, hindering members’ satisfaction or team productivity [72,73,74]. In addition, past studies showed that relationship conflict is likely to reduce team commitment and increase turnover intention [75,76]. In light of these findings, we wanted to ensure that the humor of the leader still exhibits unique explainability (incremental validity) after controlling the variable of conflict with the leader as recognized by the follower. We measured the conflict with a leader using the seven-item scale from Xin and Pelled [77]. Sample items include “How much are personality conflicts evident between you and the leader?” and “How much tension is there between you and the leader?”

## 4. Results

Descriptive statistics and zero-order correlations for our study variables are displayed in Table 1. Affiliative humor was positively related to team commitment (r = 0.15, *p* < 0.01) and change-oriented organizational citizenship behavior (r = 0.13, *p* < 0.01). However, aggressive humor was related neither to team commitment (r = 0.01, n.s.) nor to change-oriented organizational citizenship behavior (r = −0.09, n.s.). Additionally, team commitment was positively related to change-oriented organizational citizenship behavior (r = 0.60, *p* < 0.001).

The results from our regression analyses are presented in Table 2. The dependent variables are team commitment and change-oriented organizational citizenship behavior for Models 1 through 4 and Model 5, respectively. For testing the indirect effects of leader humor styles on change-oriented organizational citizenship behavior and evaluating whether those results were conditional upon the level of leader Machiavellianism, we used the bootstrapping procedures (PROCESS, Models 4 and 7, respectively) prescribed by Preacher, Rucker, and Hayes [78].

In Hypothesis 1, we proposed that a leader’s affiliative humor increases a follower’s team commitment. Our results supported this hypothesis, indicating that a leader’s affiliative humor was positively related to a follower’s team commitment (*β* = 0.11, *p* < 0.05; Model 2). On the other hand, a leader’s aggressive humor neither decreased nor increased a follower’s team commitment (*β* = 0.12, n.s.; Model 2). Thus, Hypothesis 2 was not supported. In Hypothesis 3, we proposed that followers who report high levels of team commitment are more likely to engage in change-oriented organizational citizenship behavior. As predicted, our results show that team commitment increased change-oriented organizational citizenship behavior (*β* = 0.56, *p* < 0.001; Model 5). Thus, Hypothesis 3 was supported.

Hypotheses 4 and 5 predicted a mediating effect of team commitment in the relationship between leader humor style and change-oriented organizational citizenship behavior. Model 5 in Table 2 presents the results including the proposed mediator (team commitment) and a dependent variable (change-oriented organizational citizenship behavior) in the same model. First, we found that the effect of affiliative humor on change-oriented organizational citizenship behavior was not significant (*β* = 0.04, n.s.), pointing to the impact of the full mediation effect of team commitment in the relationship between affiliative humor and change-oriented organizational citizenship behavior. Bootstrapping results also demonstrated that, whereas the direct effect of affiliative humor on change-oriented organizational citizenship behavior was not statistically significant (direct effect = 0.040, SE = 0.058, bootstrap 95% confidence interval, CI: (−0.074, 0.153)), the indirect effect was positive and statistically meaningful (indirect effect = 0.10, SE = 0.06, bootstrap 95% confidence interval, CI: (0.014, 191)) because the CI did not include zero, providing support for Hypothesis 4. Regarding the mediation effect of team commitment in the relationship between aggressive humor and change-oriented organizational citizenship behavior, the effect of aggressive humor on change-oriented organizational citizenship behavior was negative and statistically meaningful (*β* = −0.12, *p* < 0.05; Model 5). However, as determined in our test for Hypothesis 2, aggressive humor had no effect on team commitment. Bootstrapping results also demonstrated that, whereas the direct effect of aggressive humor on change-oriented organizational citizenship behavior was negative and statistically meaningful (direct effect = −0.187, SE = 0.076, CI: (−0.337, −0.037)), the indirect effect was not statically significant (indirect effect = −0.079, SE = 0.060, bootstrap 95% confidence interval, CI: (−0.200, 0.034)) because the CI included zero. Thus, Hypothesis 5 was not supported.

**Figure 1:**
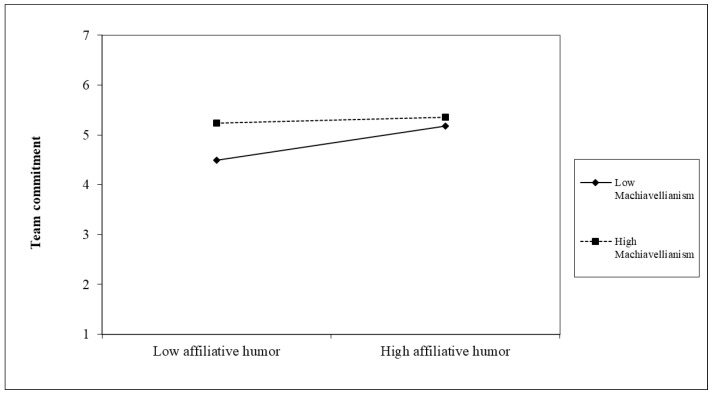
Interaction of affiliative humor and leader Machiavellianism on team commitment.

Lastly, we tested whether leader Machiavellianism moderates the indirect effect of leader humor style on change-oriented organizational citizenship behavior via team commitment. We found that the indirect effect of affiliative humor on change-oriented organizational citizenship behavior via team commitment was positive when leader Machiavellianism was low (indirect effect = 0.159, SE = 0.076, CI: (0.027, 0.318)). When leader Machiavellianism was high, however, the indirect effect was not statistically meaningful (indirect effect = 0.003, SE = 0.034, CI: (−0.066, 0.068)). The index of moderated mediation was significant (index = −0.063, SE = 0.033, CI: (−0.132, −0.004)). Hence, these results support Hypothesis 6, the conditional indirect effect of affiliative humor on change-oriented organizational citizenship behavior via team commitment. Figure 1 confirms the proposed interaction effect of affiliative humor and leader Machiavellianism on team commitment. Furthermore, the slope of the relationship between affiliative humor and team commitment was not significant when leader Machiavellianism was high (simple slope = 0.07, *t* = 0.25, n.s.), but it was positive and statistically significant when leader Machiavellianism was low (simple slope = 0.39, *t* = 2.88, *p* < 0.001).

In Hypothesis 7, we proposed the conditional indirect effect of aggressive humor on change-oriented organizational citizenship behavior via team commitment. Our results indicate that the indirect effect of aggressive humor on change-oriented organizational citizenship behavior via team commitment was statistically not meaningful when leader Machiavellianism was low (indirect effect = −0.116, SE = 0.072, CI: (−0.265, 0.016)) as well as high (indirect effect = −0.053, SE = 0.063, CI: (−0.185, 0.066)). The index of moderated mediation was also not significant (index = 0.025, SE = 0.035, CI: (−0.046, 0.095)). Thus, we found no support for Hypothesis 7.

## 5. Discussion

This study examined the relationship between leader humor and change-oriented organizational citizenship behavior via team commitment along with the moderating effect of leader Machiavellianism. Our results showed that team commitment mediates the positive relationship between a leader’s affiliative humor and the followers’ change-oriented organizational citizenship behavior. However, the indirect effect of affiliative humor on the followers’ change-oriented organizational citizenship behavior varies by leader Machiavellianism. Specifically, when leader Machiavellianism is high, the mediation effect of team commitment becomes non-significant. On the other hand, whereas a leader’s aggressive humor is negatively related to followers’ change-oriented organizational citizenship behavior, team commitment did not mediate that relationship. One of the possible explanations for our findings could be the characteristics of our sample. The effect of leader humor is affected by power relations [24]. Because the military is s highly authoritarian setting placing importance on a hierarchy, aggressive humor is often taken for granted as a prototypical leader humor style by followers [79]. Hence, in the military setting, leaders who use positive humor at work are benefitted by being rated as more competent leaders by their followers because they are perceived as being more open-minded compared to others [80]. Hence, unlike affiliative humor, it seems that the leaders’ aggressive humor has a direct effect on the followers’ change-oriented organizational citizenship behaviors, not an indirect effect via team commitment. In a similar vein, we conducted a supplementary analysis and found that the interaction effect of aggressive humor and leader Machiavellianism on the followers’ change-oriented organizational citizenship behavior is not statistically significant (*β* = 0.01, n.s.), implying that, when leaders use aggressive humor in the military setting, it is likely that followers take it for granted and thus the extent to which leaders are Machiavellian does not have an effect on whether they engage in change-oriented organizational citizenship behaviors.

### 5.1. Theoretical Implications

First, our findings contribute to the literature on leader humor by demonstrating its relationship with the followers’ change-oriented organizational citizenship behavior. To the best of our knowledge, the relationship between leader humor and followers’ change-oriented organizational citizenship behavior has not been empirically examined. Although change-oriented organizational citizenship behavior is closely related to innovation-relevant behaviors at work, which are also critical for the innovation, it differs in that it is not included as a part of the organization’s formal reward system [81]. Behaviors beyond one’s formal job requirements are vital for innovation because followers who engage in change-oriented organizational citizenship behaviors are more willing to support their organizations’ success [82]. Thus, our findings shed light on the role of humor in energizing innovation in the workplace.

Second, our findings offer an explanation for how leader humor could strengthen or weaken the followers’ change-oriented organizational citizenship behavior by demonstrating that team commitment mediates the positive link between a leader’s affiliative humor and change-oriented organizational citizenship behavior. Specifically, supporting the relational model [24] and wheel model of humor [58], our results showed that followers develop positive attitudes toward their teams (i.e., team commitment) as a result of experiencing leaders’ affiliative humor which, in turn, facilitates their change-oriented organizational citizenship behavior. These findings enrich our knowledge of how leader humor affects followers’ extra-role behaviors that are critical for the innovation.

Third, our empirical results contribute to the humor literature by examining the roles of leader Machiavellianism as a moderator in the relationship between leader humor and change-oriented organizational citizenship behavior. Specifically, given the calls for research on the role of potential moderators in better explaining the effect of humor at work [10], our findings enrich our understanding of how individual-difference factors in leader humor lead to different outcomes. In addition, compared to the robust research on the relationship between big five personality and humor, there is a relative scarcity of empirical data about the role of dark triad personality in explaining humor in the workplace. Our results showed that, whereas leader Machiavellianism is not related to affiliative humor, there is a positive relationship between leader Machiavellianism and aggressive humor, thus providing a constrictive replication of past studies [83,84].

### 5.2. Practical Implications

Our study provides practical insights into managers. Our findings suggest that humor, especially affiliative humor, needs to be included as one of the competencies that organizational leaders should possess. Humor has been regarded as the crux of fun management. For instance, in the case of Southwest Airlines in the United States, a leader’s humor was actively used in management strategies and leadership approaches to elicit positive performance and members’ commitment [85]. Our results revealed that the extent to which followers are committed to their teams—and even engage in change-oriented organizational citizenship behavior—is largely determined by the type of leader humor. As such, our study suggests that, to promote the innovation through the increase in the followers’ change-oriented organizational citizenship behavior, leaders should use positive forms of humor (e.g., affiliative humor) rather than negative types of humor (e.g., aggressive humor). It may be advisable for organizations, especially those in high need of innovation at work, to start with a small implementation of a leader’s proper use of humor, rather than investing their financial resources, to facilitate the followers’ change-oriented organizational citizenship behavior. Specifically, organizations could encourage leaders to use affiliative humor by creating an organizational climate in which positive humor is well accepted and by presenting guidelines to prevent the use of aggressive humor. Furthermore, our findings suggest that when leaders express affiliative humor at work, they should ensure that this humor is perceived by their followers as evidencing their genuine support toward them. To do this, in addition to their expression of affiliative humor at work, leaders may need to provide followers with adequate support on a continuous basis.

### 5.3. Limitations and Future Research Directions

Our study is not without limitations. First, we used measured leader humor based on leaders’ self-ratings following Martin et al. [9]. However, we cannot rule out the possibility that leaders and followers may have different perceptions of leader humor. For this reason, some studies have measured the leader’s humor using the followers’ perceptions [31]. Thus, future research could enrich our study’s findings by measuring the leader’s humor from at least two different perspectives.

Second, of the three personality traits in the dark triad (Machiavellianism, narcissism, and psychopathy), we tested only the role of Machiavellianism. Past studies showed that the different dark-triad traits could have varying impacts on the use of humor [83,84]. Future research could expand our findings by exploring the roles of the other two dark-triad traits in facilitating or hindering followers’ change-oriented organizational citizenship behavior.

Third, our model paid its attention explicitly to followers’ change-oriented organizational citizenship behaviors. Thus, although the relationship between leader humor and the followers’ organizational citizenship behaviors are well known [11,15,56], we do not know whether team commitment mediates the relationships. In addition, if so, we have no knowledge whether the mediated relationships vary by a leader’s Machiavellianism either. Hence, future research could benefit by expanding our model to followers’ organizational citizenship behaviors.

Fourth, the military-based data we used could raise a generalizability issue. Compared to that in most private organizations, the Army culture is highly authoritarian and places importance on maintaining a hierarchy. The humor exchanged within organizations reflects such cultural characteristics. In fact, many forms of humor in the Army are often based on the negative reality of military life [86,87]. Unlike followers in private organizations, soldiers may exhibit less resistance to their leaders’ aggressive humor. Thus, future research could utilize our model in examining followers in private organizations. In addition, our data are cross-sectional in nature, which does not allow us to make inferences about the causality of the study variables in the model. For more rigorous findings, future research could apply our model using a longitudinal research design.

## Figures and Tables

**Table 1:** Means, Standard Deviations, and Correlations.

Variables	Mean	SD	1	2	3	4	5	6
1. Conflict	2.07	1.10	(0.96)					
2. Affiliative humor	5.14	0.89	−0.09	(0.83)				
3. Aggressive humor	2.93	0.76	−0.00	−0.13 **	(0.71)			
4. Machiavellianism	3.66	1.23	0.00	0.06	0.47 ***	(0.79)		
5. Team commitment	5.06	1.27	−0.41 ***	0.15 **	0.01	0.19 ***	(0.96)	
6. COCB	4.50	1.22	−0.29 ***	0.13 **	−0.09	0.11 *	0.60 ***	(0.93)

Note. * *p* < 0.05. ** *p* < 0.01. *** *p* < 0.001. COCB = Change-oriented organizational citizenship behavior. Reliabilities are on the diagonal in parentheses.

**Table 2:** Regression Results.

	Team Commitment				COCB
Variables	Model 1	Model 2	Model 3	Model 4	Model 5
Conflict	−0.41 ***	−0.40 ***	−0.41 ***	−0.40 ***	−0.05
Affiliative humor		0.11 *	0.08	0.10 *	0.04
Aggressive humor		0.02	−0.10	−0.09	−0.12 *
Machiavellianism			0.23 ***	0.23 ***	0.06
Affiliative humor × Machiavellianism				−0.10 *	−0.07
Aggressive humor × Machiavellianism				0.04	−0.02
Team commitment					0.56 ***
Adjusted *R*^2^	0.17	0.18	0.22	0.22	0.37
Δ*R*^2^		0.01	0.04	0.01	
Overall *F*	78.14 ***	28.18 ***	27.19 ***	19.21 ***	32.29 ***
Δ*F*		2.82	19.96 ***	2.74 **	

Note. * *p* < 0.05. ** *p* < 0.01. *** *p* < 0.001. COCB = Change-oriented organizational citizenship behavior.

## Data Availability

The data presented in this study are available on request from the corresponding author.
